# Impact of stakeholder perspectives on cost-effectiveness estimates of four specialized nutritious foods for preventing stunting and wasting in children 6–23 months in Burkina Faso

**DOI:** 10.1186/s12937-020-00535-x

**Published:** 2020-02-27

**Authors:** Ye Shen, Ilana R. Cliffer, Devika J. Suri, Breanne K. Langlois, Stephen A. Vosti, Patrick Webb, Beatrice L. Rogers

**Affiliations:** 1 Friedman School of Nutrition Science and Policy, Tufts University, 150 Harrison Ave, Boston, MA 02111 USA; 2 Department of Nutritional Sciences, University of Wisconsin-Madison, Madison, WI USA; 3 Department of Agricultural and Resource Economics, University of California Davis, Davis, California USA

**Keywords:** Cost-effectiveness, Stunting, Wasting, Infant and young children, Supplementary feeding, Opportunity cost, Caregiver, West Africa

## Abstract

**Background:**

Multiple specialized nutritious food options are programmed for supplementation in humanitarian and development settings. However, comparative cost-effectiveness evidence is lacking, let alone incorporation of perspectives from uncompensated stakeholders. A Burkina Faso trial evaluated the cost-effectiveness of Corn Soy Blend Plus w/ oil (CSB+ w/oil, reference arm), Corn Soy Whey Blend w/oil (CSWB w/oil), Super Cereal Plus (SC+), and Ready-to-Use Supplementary Food (RUSF) in reducing stunting and wasting among children 6–23 months old. This paper presents cost-effectiveness findings from multiple stakeholders’ perspectives, including caregivers and program volunteers.

**Methods:**

An activity-based costing with ingredients approach was used to summarize cost of the 18-month-long blanket supplementary feeding for each enrolled child (in 2018 USD). Time data were collected using self-reported and observational instruments. Cost-effectiveness relative to CSB+ w/oil assessed incremental cost per enrolled child against incremental outcomes: prevalence of stunting at 23 months of age and number of months of wasting. Two combined perspectives were compared: *program* (donor, implementer, and volunteer) versus *program and caregiver* (adding caregiver).

**Results:**

A total of 6112 children were enrolled. While similar effectiveness was found in three arms (CSWB w/oil was less effective), costs differed. Product cost and caregiver time to prepare study foods were major drivers of cross-arm cost differences from the respective combined perspective. The two major drivers were used to construct uncertainty ranges of cost per enrolled child from *program and caregiver* perspective: $317 ($279- $355) in CSB+ w/oil, $350 ($327- $373) in CSWB w/oil, $387 ($371- $403) in RUSF, and $434 ($365- $503) in SC+. Cost from *program and caregiver* perspective was a substantial increase from *program* perspective. CSB+ w/oil was most cost-effective in reducing stunting and wasting, and this main finding was robust to changing perspectives and all corresponding sensitivity analyses when uncompensated time was valued at minimum wage ($0.36/h). The break-even point for uncompensated time valuation is >$0.84/h, where RUSF became the most cost-effective from the *program and caregiver* perspective. Relative cost-effectiveness rankings among the other three arms depended on choice of perspectives, and were sensitive to values assigned to product cost, international freight cost, opportunity cost of time, and outcomes of a hypothetical control. Volunteer opportunity cost did not affect arm comparisons, but lack of compensation resulted in negative financial consequences for caregivers.

**Conclusions:**

Evaluating cost-effectiveness by incorporating uncompensated stakeholders provided crucial implementation insights around nutrition products and programming.

**Trial registration:**

Trial registration number: NCT02071563.

Name of registry: ClinicalTrials.gov

URL of registry: https://clinicaltrials.gov/ct2/show/NCT02071563?type=Intr&cond=Malnutrition&cntry=BF&draw=2&rank=9

Date of registration: February 26, 2014.

Date of enrollment of first participant: July 2014.

## Background

In 2017, there were an estimated 151 million children worldwide under five who were stunted (< − 2 standard deviations in height-for-age) and 51 million who were wasted (< − 2 standard deviations in weight-for-height) [1]. The first 1000 days of life, which begins in utero and continues into the first two post-natal years, has been identified as a critical window of opportunity to prevent such manifestations of undernutrition [2, 3], thereby avoiding long-term consequences to human capital and societal development [4, 5].

The 2013 Lancet series identified ten key nutrition-specific interventions with evidence of effectiveness. Scaling up just these ten interventions would cost $9.6 billion annually [6]. More than half of the estimated $9.6 billion would be allocated to food supplementation programs for two target recipient groups: pregnant women, and young children [7]. It was estimated that for every dollar invested to reduce stunting through these nutrition interventions in selected high burden countries in Sub-Saharan Africa, the economic returns ranged from US$4 to US$24 [8]. However, national governments and donor agencies have limited resources to dedicate to these important tasks. It is therefore critical that decisions on funding allocations be based on rigorous evidence of what works best, and at what cost. Incorporating economic analyses into studies of the effectiveness of programming for nutrition is a high priority.

There have been many calls for high-quality and timely publications of costing and cost-effectiveness to generate actionable evidence [9], especially as this pertains to food assistance [10], prevention of acute malnutrition [11], and implementation science relating to nutrition more broadly [12–14]. While several efficacy and effectiveness trials have evaluated various Specialized Nutritious Foods (SNFs), products formulated with macronutrients and micronutrients, such as lipid-based nutrient supplements (LNS) and fortified blended flours (FBF) that are commonly used in programs that seek to prevent or that treat undernutrition. In preventive supplementary feeding programs [15], past research has focused little on the cost-effectiveness of products used or the ways in which products are delivered.

Furthermore, many stakeholders are involved in different aspects of supplementary feeding programs. Funders/donors provide financial resources to the programs and may manage upper levels of supply chains, including product procurement and international freight. Implementers are involved in the entire supply chain and in the implementation of the supplementary feeding program. Volunteers, often recruited by implementers from the local communities, operate some important program activities. Recipients and/or their caregivers put in extra time to participate in programs. These stakeholders have different perspectives regarding the costs of supplementary feeding programs. Choice of costing perspective reflects the burden of costs borne by different stakeholder group, each of which may play important roles in program performance and sustainability. The inclusion or exclusion of perspectives could affect the cost-effectiveness comparisons across interventions. Furthermore, direct and indirect costs to households of accessing child nutritional products and services can be especially higher in impoverished and marginalized populations [16]. However, perspectives of volunteers and food aid recipients/caregivers are rarely included in economic analyses of supplementary feeding programs in resource-poor settings, pointing to major gaps in understanding opportunity costs associated with such programs and likely underestimates of overall program costs. A 2009 review of management of acute malnutrition in resource-poor settings suggested that “a formal cost-effectiveness analyses including clinic staff time and household opportunity costs has not yet been reported” to compare different SNF options (especially between ready-to-use foods and fortified blended flours) for targeted supplementary feeding to treat moderate acute malnutrition [17]. A decade later, such cost-effectiveness analysis is still lacking to support product choices in all types of supplementary feeding programs.

The research team conducted a field trial that evaluated the relative cost-effectiveness of programing four types of SNFs to prevent stunting and wasting among children 6–23 months in an existing blanket supplementary feeding program in the Center-North region of Burkina Faso. This region has experienced high rates of undernutrition, approximately 29% prevalence of stunting and 25% prevalence of wasting among children under five years of age 2010 [18], and a functioning prevention program “Victoire sur la Malnutrition” (ViM) was in existence since 2011 to address these issues. This paper presents cost-effectiveness results from the perspectives of multiple stakeholders, and reports sensitivity analyses of cost-effectiveness estimates corresponding to each perspective. It complements all effectiveness findings and the primary cost-effectiveness results from a single program perspective reported elsewhere [19].

## Methods

### Study design and setting

Between 2014 and 2016, a blanket supplementary feeding program called “Victoire sur la Malnutrition” (ViM) distributed food and measured young children (~ 6 to ~ 23 months from age) monthly. Four regions in rural Sanmatenga Province of Burkina Faso were randomly assigned to one of four intervention arms: Corn Soy Blend Plus with fortified vegetable oil (CSB+ w/oil, reference arm), Corn Soy Whey Blend with fortified vegetable oil (CSWB w/oil), Super Cereal Plus (SC+), or Ready-to-Use Supplementary Food (RUSF). The four regions are comparable across important characteristics as described elsewhere [19].

The reference arm, CSB+ w/oil, was standard-of-care for the USAID-funded ViM program. SC+ and RUSF are SNFs commonly programmed by other international agencies, and CSWB is an experimental product that added whey protein concentrate to the CSB+ formulation [20]. As described in Table 1, differences in product specifications (e.g. formulation and packaging) and associated programming (e.g. storage, repackaging, and food preparation) carry implications for varying costs and effectiveness among the study foods. Product packaging for all study foods was consistent with common USAID programming.

**Table 1:** Comparison of Intervention Arms Based on Differences in Programming of Study Foods

	CSB+ w/oil (*n* = 1312)	CSWB w/oil (*n* = 1255)	SC+ (*n* = 1324)	RUSF (*n* = 1313)
Ration size: kcal/day	500	500	500	500
Ration size: g/day	75 (flour) + 22.4 (oil)	75 (flour) + 22.4 (oil)	120	92
SNF type	FBF + FVO	FBF + FVO	FBF	LNS
Programmed or experimental	Programmed	Experimental	Programmed	Programmed
Packaging specification	25 kg bag (flour) + 4 L can (oil)	25 kg bag (flour) + 4 L can (oil)	1.22 kg bag	92 g sachet
In-country (Burkina Faso) extra handling steps	Repackaging of flours into 2.25 kg bags; reconditioning of oil with damaged cans; pouring of oil into caregivers’ containers at distribution	Repackaging of flours into 2.25 kg bags; reconditioning of oil with damaged cans; pouring of oil into caregivers’ containers at distribution	None	None
Preparation and feeding	Cook with boiling water	Cook with boiling water	Cook with boiling water	Ready to use
Ingredients	Flour: corn, soybeans, vitamin/mineral premix; oil: vegetable oil fortified with Vitamin A & D	Flour: corn, soy flour, whey protein, vitamin/mineral premix; oil: vegetable oil fortified with Vitamin A & D	Corn, soybeans, dried skim milk powder, sugar, soybean oil, vitamin/mineral premix	Oilseeds, peanuts, pulses, cereals, sugar, dairy protein, vegetable oil, vitamin/mineral premix

The study protocol was approved by Tufts University Institutional Review Board and Ethics Board of the Ministry of Health, Burkina Faso, and registered with ClinicalTrials.gov [NCT02071563] [21]. Details on the population, overall study methods, and programmatic setting were described elsewhere [19].

### Stakeholders’ perspectives

**Fig. 1:**
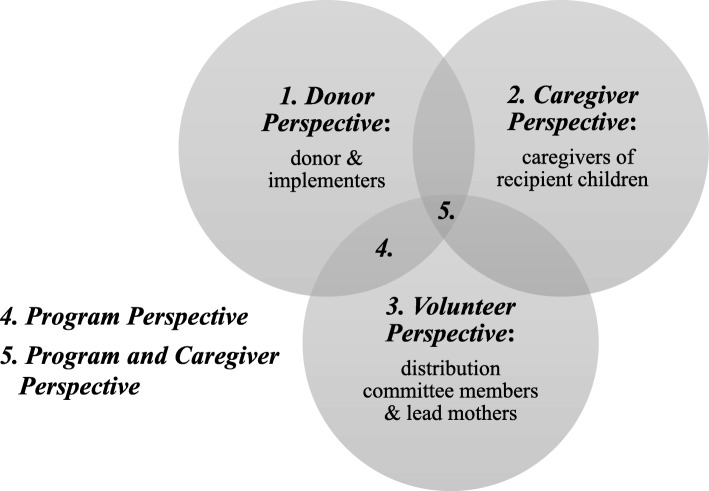
Five costing perspectives and corresponding stakeholders

The donor (USAID), implementers (ACDI/VOCA and Save the Children), volunteers (distribution committee members and lead mothers), and caregivers of the recipient children were the main stakeholders involved in this program, and the research team constructed five costing perspectives based on these stakeholders (as shown in Fig. 1**)**. Financial resources paid by the donor covered costs incurred by the donor and implementers, and therefore represent program costs from the *donor* perspective, excluding the opportunity cost of all uncompensated time. Uncompensated time was captured in the costing perspectives of *caregiver* as well as *volunteer* groups. Relative cost-effectiveness was compared between two combined perspectives: *program* perspective, including costs incurred by the donor, implementers, and community volunteers; and *program & caregiver* perspective, adding *caregiver* perspective to that of the *program* perspective.

Throughout the rest of the paper, “base-case scenario” refers to a set of defined values (as described below) assigned to cost parameters corresponding to each perspective in the estimation of the base-case summary cost by arm. This term is used to differentiate from the values assigned to selected parameters in the sensitivity analyses. This paper reports cost and cost-effectiveness results from the perspectives of multiple stakeholders with sensitivity analyses for each perspective. Results of the base-case scenario from *program* perspective were reported as the primary cost-effectiveness finding elsewhere [19].

### Cost data collection and categorization

An activity-based costing with ingredients approach (ABC-I) was used for estimating all costs associated with each arm [22–24]. Major activities along food procurement, supply chain, and the blanket supplementary feeding program were identified, and the information on quantities and prices to value all resources (ingredients) needed was assigned to each activity to calculate costs. Table 2 shows the nine cost components/activities and their corresponding definitions and data sources. The ViM program had been ongoing before the trial began, so start-up costs for the program could not be captured. This does not affect the comparative cost-effectiveness of the study foods, but the total cost per enrolled child may be somewhat underestimated.

**Table 2:** List of Cost Components with Definitions and Data Sources

Cost Component/ Activity	Included Stakeholder Perspective(s)	Definition	Data Source
Food Product	Donor Perspective	Cost of the specific specialized nutritious food and additional fortified oil (if applicable)	Billing records from Didion Milling, Challenge Dairy, + Edesia; historical data from USAID/Food For Peace, and realistic quote from Didion Miling
International Freight	Donor Perspective	Cost of international shipping from USA to Ouagadougou, Burkina Faso	Billing records from ACDI/VOCA and realistic quotes from BKA Logistics
Inland Transportation	Donor Perspective	Cost of transportation from the main warehouse in Ouagadougou to Food Distribution Points in the study site	Billing records from Etablissement Kafando Mahamadi (EKM) + Save the Children
Storage	Donor Perspective	Cost of storing the foods at the main warehouse in Ouagadougou including space, labor, fumigation, destruction, utilities, commodity handling, lab testing and analysis, other services and supplies	Warehouse documents and accounting records – ACDI/VOCA
Repacking (CSB+ and CSWB ONLY)	Donor Perspective	Cost of repacking the 50 kg bags of CSB+ and CSWB into 2.25 kg bags (labor and materials)	Warehouse documents and accounting records – ACDI/VOCA
Reconditioning (Fortified Vegetable Oil ONLY)	Donor Perspective	Cost of reconditioning fortified oil that were leaking from the cans (labor and materials)	Warehouse documents and accounting records – ACDI/VOCA
Distribution	Donor Perspective; Volunteer Perspective	Cost of labor (including staff cost and opportunity cost of volunteer distribution committee members) and fixed supplies	Observations at food distribution points and accounting records– ACDI/VOCA
Administrative and Overhead Costs	Donor Perspective; Volunteer Perspective	Cost labor (including implementation partners’ staff cost and opportunity cost of lead mothers involved in SBCC), training, and administrative overhead costs	Accounting records and interviews with ACDI/VOCA and Save the Children
Caregiver Cost	Caregiver Perspective	Caregiver spending in transportation and opportunity cost of caregivers’ time participating in the program	In-home observations, observations at food distribution points, and study surveys with caregivers

*ACDI/VOCA* Agricultural Cooperative Development International/ Volunteers in Overseas Cooperative Assistance, *CSB+* Corn Soy Blend Plus, *CSWB* Corn Soy Whey Blend, *SBCC* Social and Behavior Change Communication

Cost data were all collected as part of the blanket supplementary feeding program evaluated by the study, with the exception of food product prices and international freight costs. Study foods procured for the trial were produced and shipped at varying scales which differentially affected study-incurred product and international freight costs. CSB+ and oil had been programmed through ViM several years prior to the study. In contrast, SC+, RUSF and CSWB were procured for the study at a much smaller scale than for a typical USAID supplementary feeding program. Therefore, in order to compare product and international freight costs among the four arms at the same scale from the same data source if possible, realistic product prices for CSB+, oil, SC+, and RUSF were estimated using USAID Food for Peace’s transaction-level data from Fiscal Year 2014 to 2016 [25]. CSWB was an experimental product proposed during Phase I of the FAQR project [20], and had never been produced prior to the study. Therefore, CSWB product price was derived from a quote based on production of ≥500 metric ton (MT) by Didion Milling Inc., the USAID supplier who produced the CSWB flour for the study. Realistic international freight cost per MT was estimated for shipping from US to Ouagadougou, Burkina Faso based on product-specific shipping cost for maximum loading quantities per 20′ container (quotes provided by USAID freight forwarder, BKA Logistics LLC). The research team accounted for percent product losses recorded during international freight, inland transportation, storage, and repacking by adjusting all affected cost components. Even though losses likely also occurred during distribution, such data were too unreliable for inclusion.

ViM program operations relied on unpaid food distribution committee members who distributed study foods and lead mothers who disseminated Social Behavior Change Communication (SBCC). Lead mothers were mothers selected from the communities who served as group leaders in SBCC activities to teach program participants about the purpose, use, and consumption of the study foods. Caregivers of recipient children also spent time collecting, preparing, and serving the study foods. To capture time use, the research team conducted 48 distribution observations of volunteers and caregivers at food distribution points (one-day observation per site), 209 in-home observations of caregivers and children (12-h-per-day observation over four days per household), and 1612 interviews with caregivers. Lead mother time-use was estimated based on information provided by implementation partners. Total time spent in each activity was multiplied by an hourly value of time to estimate opportunity costs. In the base-case scenario, the research team used the hourly minimum wage of $0.36 (162 CFA in 2006) for agricultural workers mandated in Burkina Faso law, due to study’s rural setting [26]. In FBF arms (CSB+ w/oil, CSWB w/oil, and SC+), caregivers’ time attributed to study food preparation accounted for other major concurrent activities. Inverse weights (1 for food preparation shared with no other activity, 1/2 for one other activity, 1/3 for two other activities) were applied to each observed meal preparation occasion. The average time attributed to study food preparation per meal was then multiplied by average number of meals prepared to calculate total time spent in preparing study flours per month for each arm.

Aside from time data from caregivers’ perspective, the research team collected quantitative data during interviews with caregivers about their monetary spending for transportation to and from food distribution sites. Additionally, the research team collected qualitative data from focus groups about caregivers’ experiences with the food distribution process. Details on qualitative research methods for the overall trial can be found elsewhere [27].

All cost results are reported in 2018 United States dollars (USD), unless specified otherwise. Costs incurred in Burkina Faso’s currency, the West African franc (CFA), were first converted to USD of the same incurred year based on corresponding annual exchange rate of that year [28], and then converted to USD in 2018 (analysis year) adjusting for United States annual inflation rates measured by a GDP implicit deflator [29].

### Summary cost measures

The primary unit of measure was total cost per enrolled child. To calculate this, each cost component was first summarized into one of the three composite measures: cost per MT for each of the five products, cost per monthly ration per arm, or cost per enrolled child per arm. Then, cost components summarized in cost per MT and cost per monthly ration were converted into cost per enrolled child. All cost components were then added together to obtain total cost per enrolled child for each arm:

$$ Cost\  per\ {monthly\ ration}_{per\  arm}=\sum Cost\  per\ {MT}_{per\  product}\times Quantity\ (MT)\  per\ {monthly\ ration}_{per\  product} $$

$$ Cost\  per\ {enrolled\ child}_{per\  arm}= Cost\  per\ {monthly\ ration}_{per\  arm}\times \mathrm{A} verage\ number\ of\ monthly\ rations\ collected\  per\ {enrolled\ child}_{per\  arm} $$

Each “enrolled child” is defined as having received at least one 500-kcal ration during the intervention period. Because average number of monthly rations collected varied slightly by whether the sample included those who were lost to follow up or not, the sample used to calculate cost per enrolled child corresponded to each effectiveness model (explained below) in the cost-effectiveness analysis.

Top drivers of cost differences across arms were chosen to construct uncertainty ranges of cost corresponding to relevant perspectives. Uncertainty ranges for summary estimates of cost, by study arm, from the *program* perspective (as well as from *donor* perspective) were constructed based on one standard deviation (SD) above and below the three-year average (2014–2016) USAID product price data for all study foods, except for experimental CSWB. Uncertainty ranges for the *caregiver* perspective were constructed based on one SD above and below the mean value of time for preparation per meal for the three FBF arms, adjusted for concurrent activities as noted previously. Uncertainty ranges from the *program and caregiver* perspectives included both of the cost ranges described above.

### Effectiveness outcomes

The primary outcomes of the trial were defined a priori as: 1) the estimated prevalence of stunting (length-for-age z-score (LAZ) < − 2) at end-line (between age 22.9 and 23.9 months of age) using multivariable logistic regression; and 2) the estimated number of months of wasting (weight-for-height z-score (WHZ) < − 2) out of 18 possible measurement periods using multivariable negative binomial regression. The marginal mean effects of both outcomes with 95% confidence intervals (CIs) were calculated based on predicted probabilities in each arm, adjusted for predefined individual, household and community level covariates. The covariates in these multivariable models included age, sex, maternal age, wealth, baseline anthropometric status in z scores, twin status, caregiver education, ethnicity, number of children < 5 in the household, household food insecurity, illness in the last 2 weeks, seasonality, total distributions received, village level access to: water, sanitation, market, phone service, road, public transport, transport methods from the village, pharmacy, health center, and health agents. The CIs were used to construct the uncertainty ranges for effectiveness outcomes by arm.

Children who did not have a measurement between 22.9 and 23.9 months were defined as lost-to-follow-up (LTFU) for stunting, and the primary statistical model for stunting excluded LTFU. The wasting model counting monthly measurements did not define LTFU, but rather adjusted for missed measurements in the analysis. More detail about effectiveness data collection, variable selection and modeling procedures is available elsewhere [19].

### Cost-effectiveness analysis

Using methods common in other cost-effectiveness studies [30, 31], the research team obtained incremental cost and effectiveness measures:

$$ Incremental\ cost\  per\ {enrolled\ child}_{per\  arm}= Cost\  per\ {enrolled\ child}_{per\  arm}- Cost\  per\ {enrolled\ child}_{CSB+w/ oil\  arm} $$

$$ {Incremental\ effectiveness}_{per\  arm}=\kern0.5em Adjusted\ stunting\ or\ wasting\ outcome\  per\ {enrolled\ child}_{per\  arm}- Adjusted\ stunting\ or\ wasting\ outcome\  per\ {enrolled\ child}_{CSB+w/ oil\  arm} $$

**Fig. 2:**
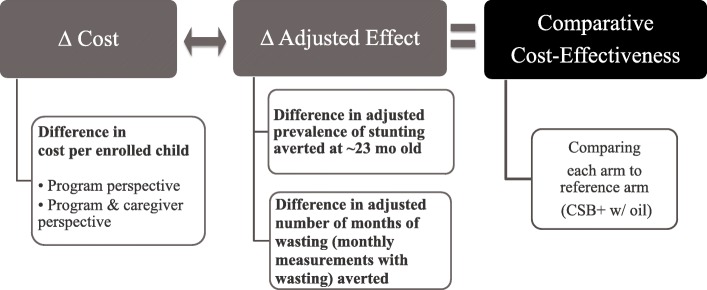
Cost-effectiveness analysis methods to compare across arms

Incremental cost per enrolled child was then linked with the specified incremental effectiveness (adjusted number of months of wasting measurements per child and adjusted prevalence of stunting at end-line), as depicted in Fig. 2. Incremental costs and incremental effectiveness results obtained from each analysis with the previously described uncertainty ranges were plotted into an incremental cost-effectiveness plane [32]. When an intervention is both cost-saving and significantly more or equally effective compared to the reference arm, this intervention is called “dominant.” When an intervention is both more costly and significantly less or equally effective compared to the reference arm, this intervention is called “dominated” [33]. However, if an arm was found to be neither “dominant” nor “dominated,” it would be necessary to calculate the Incremental Cost-Effectiveness Ratio (ICER) obtained from dividing incremental cost by incremental primary effectiveness outcomes. In this study, the ICERs would be incremental cost per additional case of stunting averted and incremental cost per additional month of wasting averted.

### Sensitivity analyses

For each of the respective costing perspectives, additional sensitivity analyses of cost were conducted for hourly valuation of time used in caregiver opportunity costs and for study-incurred versus realistic estimates of product prices and international freight costs. Multiple models were also developed to assess the effects on estimated costs of LTFU cases for stunting. As simulation modeling results showed agreement with findings from the primary stunting effectiveness model [19], the research team did not report additional cost-effectiveness analyses by different handling of LTFU when modeling stunting at end-line in this paper.

Lastly, the trial did not have a control arm due to ethical considerations, and the purpose of the study was to compare among the four foods. Therefore, absolute magnitude of cost-effectiveness for each intervention arm (compared to a non-intervened control group from the same population) could not be evaluated. If a control group had worse outcomes than at least one of the four intervention arms, the extent to which the control group had worse outcomes could affect the absolute differences in cost to achieve one unit of effectiveness among the intervention arms and could affect intervention comparisons. In order to provide a sense for absolute magnitude of stunting reduction in this study, the research team used a range of possible values of stunting prevalence at ~ 23 months for a hypothetical no intervention group, and calculated cost per additional stunting case at end-line averted from *program* perspective for each study arm using these values. These values were constructed based on an adjusted prevalence 1, 5, 10, 15, 20, and 25 percentage point(s) more than the lowest adjusted prevalence of stunting at end-line among the four arms. The maximum value of 25 percentage points difference was determined by subtracting the unadjusted lowest prevalence (18%) of stunting at end-line among the study arms from a stunting prevalence of 42% for children 18–23 month-old from the Burkina Faso Demographic and Health Survey (DHS) in 2010 [18], as this was the best available data source. Therefore, the research team assumed that it would have been unlikely for a no-intervention group in this area to have more than 42% stunting prevalence at 18–23 months. The region-specific stunting prevalence 29% in Centre-Nord for all children under-five from the same DHS data also falls within this constructed range.

All statistical models were fit using Stata 13.1 (StataCorp, Texas, USA). All cost, cost-effectiveness and sensitivity analyses were conducted in Microsoft Excel and R Version 3.4.1 (R Foundation, Vienna, Austria).

## Results

### Trial statistics

Details of the trial statistics related to enrollment, participant flow, and baseline characteristics have been reported [19]. Briefly, 908 of 6112 children (15%) were defined as lost-to-follow-up (LTFU) in the stunting model, while the wasting model included the full sample. Baseline prevalence of stunting and of wasting and the number of LTFU were similar across the four arms (*p* > 0.05). Average number of monthly rations collected in each arm was similar, ranging from 16.6 to 17.2 including LTFU, and from 17.4 to 18.1 excluding LTFU.

### Cost component analysis (program perspective)

**Fig. 3:**
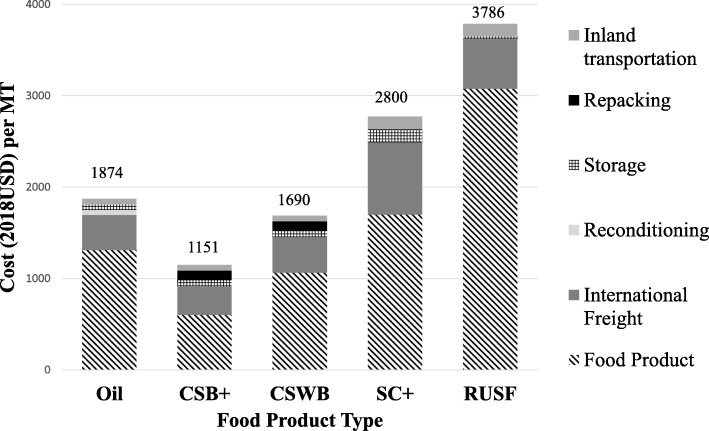
Cost per metric ton compared across products, breakdown by cost components

The six cost components along the supply chain were summarized for each of the five products into cost per MT (Fig. 3**)**. Product losses during repacking (CSB+ and CSWB only), transport, and storage amounted to about 6% of CSWB, 2% of CSB+, 0.5% of oil, 0.03% of SC+ and 0.003% of RUSF in quantity.

**Fig. 4:**
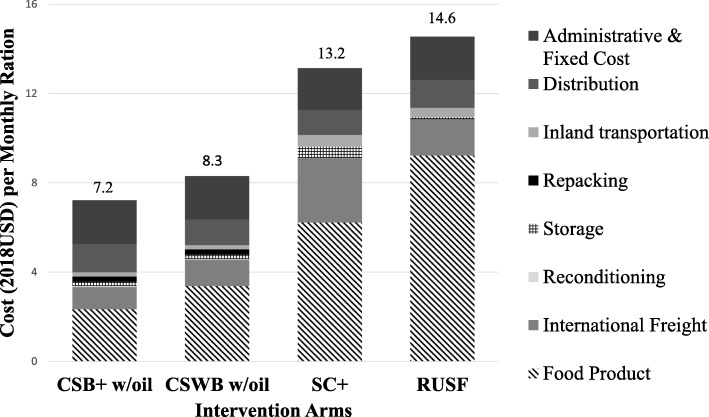
Cost per monthly ration compared across arms, breakdown by cost components

**Fig. 5:**
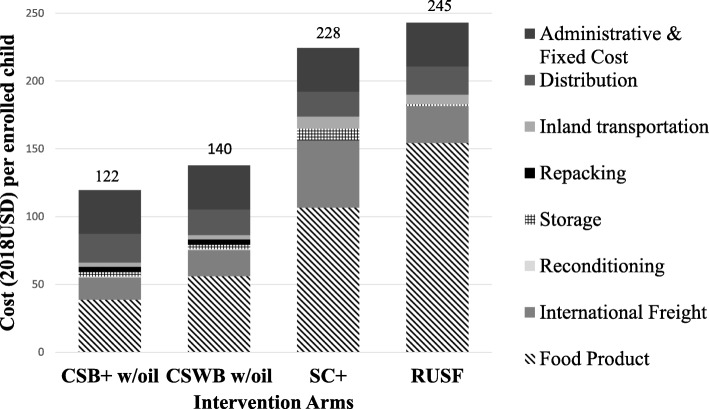
Cost per enrolled child compared across arms, breakdown by cost components

Cost components from the *program* perspective were summarized for each of the four intervention arms into cost per monthly ration (Fig. 4**)** and cost per enrolled child (Fig. 5**)**. Product cost was top cost driver for all arms, and the greatest for RUSF. SC+ had the highest international freight, inland transportation and storage costs. Despite the extra cost components for the CSB+ w/oil and CSWB w/ oil arms, CSB+ w/oil had the lowest total cost per monthly ration and per enrolled child. The most expensive arm from the *program* perspective was RUSF.

Volunteers time (Mean ± SD) was 0.48 ± 0.25 h per monthly ration with slight differences across arms for distribution committee members. Valued at $0.36/h plus $0.87 per diem, distribution committee members’ opportunity cost was about $19.8 per enrolled child. Valued at $0.36/h with Save the Children’s estimates of 5 sessions and 2 h per session led by each lead mother during the program period, lead mothers’ opportunity cost was about $0.16 per enrolled child for SBCC activities.

### Cost component analysis (caregiver perspective)

No monetary costs for transportation to and from food distribution sites were reported by caregivers in interviews. Qualitative data pointed to unanticipated occasions when distribution committee members asked caregivers to pay $0.2 (100 CFA) before collecting each monthly ration, but the research team did not design the quantitative instruments to capture the frequency of this unauthorized practice for costing. Therefore, the caregiver perspective only included the opportunity cost of caregivers’ time spent in relevant activities.

**Fig. 6:**
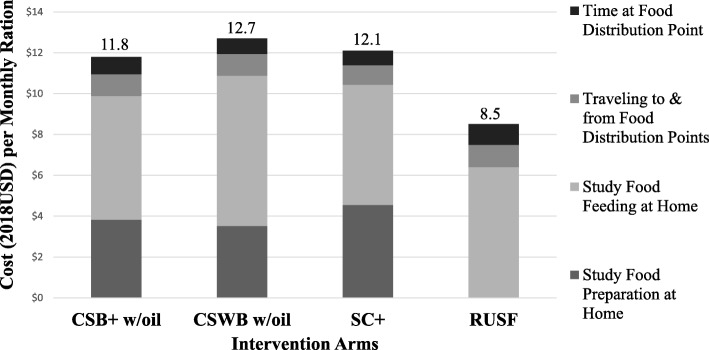
Caregiver opportunity cost per monthly ration compared across arms, breakdown by caregiver activities attributable to study foods. Hourly value of time at $0.36/h

Total caregiver opportunity cost per monthly ration in the RUSF arm was substantially less than that in the three FBF arms because RUSF did not require time to prepare before feeding (Fig. 6). Other major activities-- such as cooking other meals, bathing child, sweeping, and resting-- were observed during 68% of meal preparation occasions. Mean ± SD for observed hours attributed to study food preparation per meal was 0.15 ± 0.07 for CSB+ w/oil, 0.18 ± 0.11 for SC+, and 0.14 ± 0.05 for CSWB w/oil. For the three FBF arms, caregivers reported preparing 70–73 meals each month, amounting to 10–13 h attributed to study food preparation per monthly ration. Reported feeding time ranged from 16 to 20 h per monthly ration in the four arms. To collect each monthly ration, caregivers on average spent 3 h in traveling, and 2 h at the food distribution point with little difference across arms.

### Cost-effectiveness

**Fig. 7:**
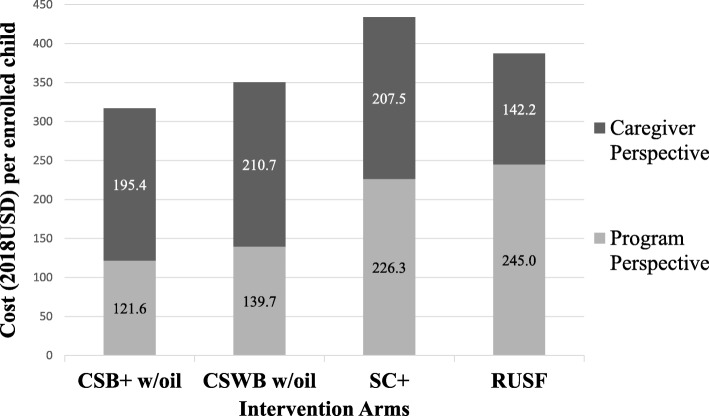
Total cost per enrolled child across arms: program perspective versus caregiver perspective. Hourly value of time at $0.36/h

Summary cost per enrolled child from each perspective and effectiveness for each arm are presented in Table 3 for the primary stunting outcome including LTFU, and in Table 4 for the primary wasting outcome excluding LTFU. As shown in the tables, *Funder* perspective which excluded volunteer opportunity cost did not differ much from the *program* perspective and did not change the relative cost rankings of the arms. Caregiver opportunity cost from *program and caregiver* perspective was a substantial addition to cost per enrolled child from *program* perspective in all arms, and especially so for the three FBF arms (Fig. 7).

**Table 3:** Summary Cost and Effectiveness Results for Adjusted Prevalence of Stunting at end-line (~23mo old) – Model Excluding Lost-To-Follow-Up (EL)

*Excluding LTFU* *n* = 5204	CSB+ w/ oil arm (n = 1312)		CSWB w/ oil arm (*n* = 1255)		SC+ arm (*n* = 1324)		RUSF arm (*n* = 1313)	
	Mean	Uncertainty Range^1^	Mean	Uncertainty Range^1^	Mean	Uncertainty Range^1^	Mean	Uncertainty Range^1^
Total Cost per enrolled child: *Base Case Program Perspective (USD 2018)*	126.6	(117.3, 135.9)	145.7	(143.1, 148.2)	236.8	(216.2, 257.5)	254.3	(237.4, 271.3)
Total Cost per enrolled child: *Caregiver Perspective (USD 2018)*	206.5	(175.4, 237.5)	222.8	(201.0, 244.5)	218.9	(167.0, 270.7)	148.3	NA
Total Cost per enrolled child: *Program and Caregiver Perspective (USD 2018)*	333.1	(292.7, 373.5)	368.5	(344.2, 392.7)	455.7	(383.2, 528.2)	402.7	(385.7, 419.6)
Total Cost per enrolled child: *Donor Perspective*^*2*^*(USD 2018)*	104.3	(94.9, 113.6)	125.6	(123.1, 128.2)	217.4	(196.7, 238.0)	232.4	(215.4, 249.3)
Adjusted Prevalence of Stunting (%) at end-line (Model EL^3^)	20.1%	(18.0, 22.2%)	27.5%*	(25.0, 30.0%)	20.3%	(18.3, 22.4%)	21.9%	(20.0, 23.9%)

^1^ Uncertainty ranges for total cost per child from *program* perspective and from *donor* perspective were constructed based on 1 standard deviation (SD) above and below the mean three-year USAID historical product cost for CSB+, RUSF, SC+, and oil. Uncertainty ranges for total cost per enrolled child from *caregiver* perspective were constructed based on 1 SD above and below the mean adjusted study food preparation time per meal for the three flour-based arms. Uncertainty ranges for *program and caregiver* perspective were the sum of the uncertainty ranges for *program* perspective and for *caregiver* perspective. Uncertainty ranges for adjusted prevalence of stunting at end-line were constructed based on 95% confidence intervals around the adjusted marginal means estimated from the respective model

^2^ Donor perspective cost per enrolled child = Program perspective cost per enrolled child – Volunteer opportunity cost per enrolled child

^3^ Adjusted Odds Ratios for each arm compared to CSB+ w/oil in the Model EL: RUSF (adj.OR: 1.02; 95% CI: 0.73, 1.44); SC+ (adj.OR: 1.21; 95%CI: 0.89, 1.66); CSWB w/oil (adj.OR: 2.07; 95%CI: 1.46, 2.94)

* *p* < 0.05 for odds ratio compared to CSB+ w/oil arm in the stunting model

**Table 4:** Summary Cost and Effectiveness Results for Adjusted Number of Months of Wasting (Measurements) – Model Including Lost-To-Follow-Up

*Including LTFU* *n* = 6112	CSB+ w/ oil arm (*n* = 1519)		CSWB w/ oil arm (*n* = 1503)		SC+ arm (*n* = 1564)		RUSF arm (*n* = 1526)	
	Mean	Uncertainty Range^1^	Mean	Uncertainty Range^1^	Mean	Uncertainty Range^1^	Mean	Uncertainty Range^1^
Total Cost per enrolled child: *Base Case Program Perspective (USD 2018)*	121.6	(112.8, 130.5)	139.7	(137.2, 142.1)	226.3	(206.7, 245.9)	245.0	(228.7, 261.2)
Total Cost per enrolled child: *Caregiver Perspective (USD 2018)*	195.4	(166.0, 224.8)	210.7	(190.1, 231.3)	207.5	(158.4, 256.7)	142.2	NA^2^
Total Cost per enrolled child: *Program and Caregiver Perspective (USD 2018)*	317.1	(278.8, 355.3)	350.4	(327.4, 373.3)	433.8	(365.0, 502.5)	387.2	(370.9, 403.4)
Total Cost per enrolled child: *Donor Perspective*^*3*^*(USD 2018)*	100.7	(91.8, 109.5)	120.5	(118.1, 122.9)	207.7	(188.1, 227.3)	224.0	(207.8, 240.2)
Adjusted Number of Months of Wasting (Number of Monthly Measurements with Wasting) per child^4^	2.4	(2.1, 2.7)	3.1*	(2.7, 3.5)	2.4	(2.1, 2.7)	2.3	(2.0, 2.5)

^1^ Uncertainty ranges for total cost per child from *program* perspective and from *donor* perspective were constructed based on 1 standard deviation (SD) above and below the mean three-year USAID historical product cost for CSB+, RUSF, SC+, and oil

Uncertainty ranges for total cost per enrolled child from *caregiver* perspective were constructed based on 1 SD above and below the mean adjusted study food preparation time per meal for the three flour-based arms

Uncertainty ranges for program and caregiver perspective were the sum of the uncertainty ranges for program perspective and for caregiver perspective

Uncertainty ranges for adjusted prevalence of stunting at end-line were constructed based on 95% confidence intervals around the adjusted marginal means estimated from the respective model

^2^ Not applicable to RUSF because uncertainty ranges for total cost per enrolled child from *caregiver* perspective were constructed based on study food preparation time which is only applicable to flour-based arms

^3^ Donor perspective cost per enrolled child = Program perspective cost per enrolled child – Volunteer opportunity cost per enrolled child

^4^ Adjusted Incidence Rate Ratios for each arm compared to CSB+ w/oil in the model: RUSF (adj.IRR: 0.93; 95%CI: 0.80, 1.09); SC+ (adj.IRR: 0.93; 95%CI: 0.80, 1.09); CSBWB w/oil (adj.IRR: 1.29; 95%CI: 1.09, 1.51)

* *p* < 0.05 for incidence risk ratio compared to CSB+ w/oil arm in the wasting model

The RUSF arm and the SC+ arm had similar effectiveness as CSB+ w/oil for primary stunting and wasting outcomes, while CSWB was significantly less effective. Compared to CSB+ w/oil, none of the other three arms had incremental costs and effectiveness that would justify the need to calculate ICERs, i.e. all costs were higher, and effectiveness outcomes were worse or not significantly different.

**Fig. 8:**
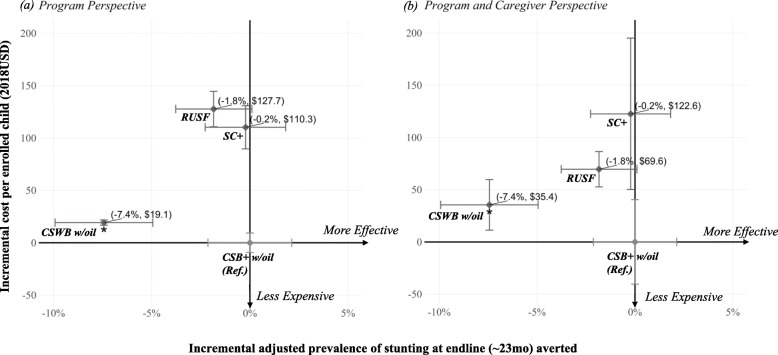
Incremental cost-effectiveness plane for stunting averted compared to CSB+ w/ oil, base-case scenario with uncertainty ranges: **a** program perspective; **b** program and caregiver perspective. Both axes were constructed comparing each of the SC+, RUSF, and CSWB w/oil arms to the reference arm CSB+ w/oil. Vertical uncertainty ranges for incremental costs from program perspective were constructed based on 1 standard deviation above and below the mean realistic product costs. Vertical uncertainty ranges for incremental costs from program and caregiver perspective additionally incorporated uncertainty in caregiver opportunity cost (1 standard deviation above and below mean adjusted study food preparation time per meal for the three flour-based arms). Horizontal uncertainty ranges for adjusted incremental effectiveness were constructed based on 95% confidence intervals around the adjusted marginal means estimated from the stunting statistical model that excluded LTFU. *p < 0.001. Data label: (point estimate on incremental effectiveness, point estimate on incremental cost)

**Fig. 9:**
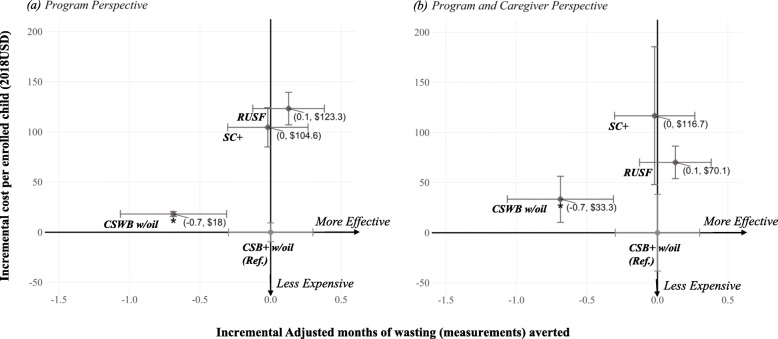
Incremental cost-effectiveness plane for wasting averted compared to CSB+ w/ oil, base case scenario with uncertainty ranges: **a** program perspective; **b** program and caregiver perspective. Both axes were both constructed comparing each of the SC+, RUSF, and CSWB w/oil arms to the reference arm CSB+ w/oil. Vertical uncertainty ranges for incremental costs from program perspective were constructed based on 1 standard deviation above and below the mean realistic product costs. Vertical uncertainty ranges for incremental costs from program and caregiver perspective additionally incorporated uncertainty in caregiver opportunity cost (1 standard deviation above and below mean adjusted study food preparation time per meal for the three flour-based arms). Horizontal uncertainty ranges for adjusted incremental effectiveness were constructed based on 95% confidence intervals around the adjusted marginal means estimated from the wasting statistical model that included LTFU. *p = 0.02. Data label: (point estimate on incremental effectiveness, point estimate on incremental cost)

Cost-effectiveness of the three arms relative to CSB+ w/oil are visualized in incremental cost-effectiveness planes to compare between the *program* perspective and the *program and caregiver* perspective (stunting: Fig. 8(a) and Fig. 8(b); wasting: Fig. 9(a) and Fig. 9(b)). From both perspectives, the CSB+ w/oil arm was the most cost-effective of the four arms for stunting averted at end-line and number of months of wasting (measurements) averted. In contrast to RUSF being the most expensive from *program* perspective, relative cost-effectiveness of RUSF from *program and caregiver* perspective substantially improved, and SC+ became the most expensive arm of the four. While uncertainty ranges of incremental cost from *program* perspective only overlapped between SC+ and RUSF, the uncertainty ranges for incremental cost from *program and caregiver* perspective widened and were closer to each other due to addition of uncertainty around caregiver time for study food preparation. However, from *program and caregiver* perspective, incremental cost range for CSB+ w/oil remained non-overlapping with cost ranges for arms of similar effectiveness (SC+ and RUSF).

### Sensitivity analyses

**Fig. 10:**
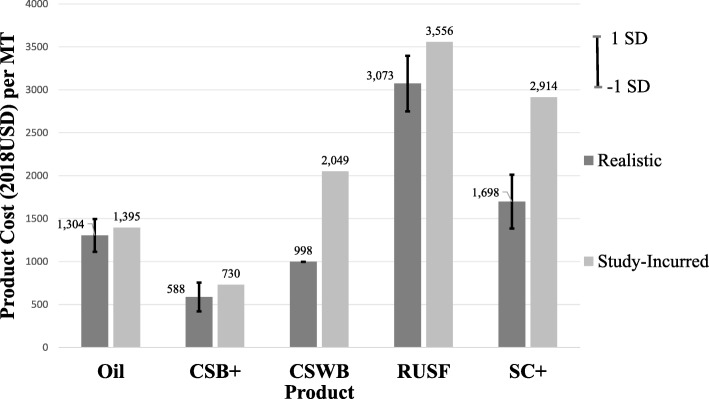
Realistic versus study-incurred product cost per metric ton. Mean and Standard Deviation (SD) of realistic prices for all products except CSWB were calculated using USAID FY14–16 three-yearhistorical data. The realistic price for CSWB was a single value without a range as it was a quote estimated at hypothetical procurement scale of >500MT provided directly by a major food aid supplier in US

**Fig. 11:**
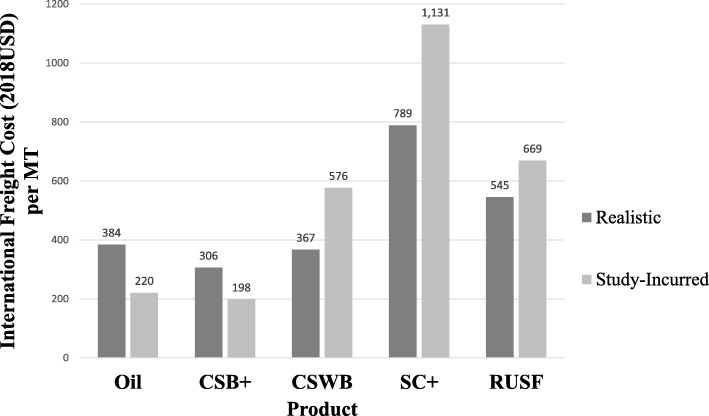
Realistic versus study-incurred international freight cost per metric ton. Realistic prices for all foods were single-value estimates as they were quotes per 20′ container provided by a major freight forwarder for USAID

Study-incurred costs per MT for product (Fig. 10) and international freight (Fig. 11) were much higher than realistic costs for CSWB, RUSF, and SC+ but similar or lower for oil and CSB+, indicating differential scales of procurement. Regarding impact of such scale variations on comparative cost-effectiveness, CSB+ w/ oil would remain the most cost-effective with widened cost differences compared to the other arms, especially SC+. While cost per enrolled child from the *program* perspective in SC+ was $105 more than in CSB+ w/oil using realistic costs, the cost difference increased to $211 using study-incurred costs for product and international freight. As a result, relative cost-effectiveness rankings from the *program* perspective switched between SC+ and RUSF, and SC+ would have been the most expensive arm from all perspectives.

As the hourly value of time used for estimating opportunity costs increased from the base-case value of $0.36/h, the RUSF arm began to have more comparable total cost per enrolled child as CSB+ w/oil from *program and caregiver* perspective. The turning point for this hourly value where RUSF began to have lower total cost per enrolled child than CSB+ w/oil was $0.84/h.

The magnitude of cost per additional case of stunting averted from *program* perspective in different hypothetical control scenarios varied substantially for all four intervention arms. Assuming only a 1 percentage point (pp) worse stunting outcome in the hypothetical control than CSB+ w/ oil, both RUSF and CSWB w/oil were dominated by no intervention, and the cost to avert one case of stunting from *program* perspective was as high as $12,320 for CSB+ w/oil and $28,653 for SC+ w/oil (Table 5). Meanwhile, when 25 pp. worse stunting outcome was assumed for the hypothetical control, cost per additional case of stunting averted dropped to $493 for CSB+ w/oil, $772 for CSWB w/oil, $902 for SC+, and $1095 for RUSF. SC+, RUSF, and CSWB w/oil changed their relative cost-effectiveness rankings with each other in the different hypothetical control scenarios, but the primary conclusion that CSB+ w/oil was the most cost-effective of the four is supported by the hypothetical control analysis.

**Table 5:** Cost-Effectiveness Analysis Based on Hypothetical Control Scenarios for Cost per Additional Stunting Case Averted^1^

	CSB+ w/oil		SC+		RUSF		CSWB w/oil	
Adjusted Prevalence Of Stunting At End-Line In Hypothetical Control	Stunting Averted, Percentage Points^2^	Cost Per Additional Stunting Case Averted^1^	Stunting Averted, Percentage Points	Cost Per Additional Stunting Case Averted^1^	Stunting Averted, Percentage Points	Cost Per Additional Stunting Case Averted^1^	Stunting Averted, Percentage Points	Cost Per Additional Stunting Case Averted^1^
21.1%	1.0	12,659	0.8	30,364	−0.8	Dominated^3^	−6.4	Dominated^3^
25.1%	5.0	2532	4.8	4955	3.2	8048	−2.4	Dominated^3^
31.1%	10.0	1266	9.8	2422	8.2	3117	2.6	5668
36.1%	15.0	844	14.8	1602	13.2	1933	7.6	1924
41.1%	20.0	633	19.8	1197	18.2	1401	12.6	1159
46.1%	25.0	506	24.8	956	23.2	1098	17.6	829

^1^ Cost per additional stunting case averted for each intervention arm was calculated as the incremental cost per child divided by incremental % stunting averted at end-line between the respective intervention arm and the specified hypothetical control value. Cost (in USD) and effectiveness (in %) results used in this analysis excluded LTFU

^2^ CSB+ w/oil had the lowest point estimate for stunting, and thus was used to construct hypothetical control values by adding 1, 5, 10, 15, 20, 25 percentage points. The maximum value of 25 percentage points was determined based on Burkina Faso DHS data in 2010

^3 “^Dominated” is an economic evaluation term to describe an intervention arm being both more expensive and less or equally effective compared the comparator (in this case the hypothetical control) which rules out the need to calculate an ICER

## Discussion

From multiple stakeholders perspectives (donor, implementers, volunteers, and caregivers), the research team evaluated the comparative cost-effectiveness of delivering monthly rations (~ 500 kcal/day) of CSB+ w/oil, CSWB w/oil, SC+ or RUSF in an 18-month blanket supplementary feeding program designed to reduce stunting and wasting among children 6–23 months of age in Burkina Faso. In all sensitivity analyses from both combined perspectives, the current standard-of-care, CSB+ w/oil, was found to be the most cost-effective of the four arms when valuing uncompensated time at $0.36/h. When assuming the value of uncompensated caregiver time to be higher than $0.84/h, RUSF would have sufficiently low caregiver opportunity cost to become the most cost-effective from the combined *program and caregiver* perspective. While the primary conclusion about CSB+ w/oil was quite robust, a few sensitivity analyses and the different perspectives did affect relative cost-effectiveness comparisons among the trial arms. To the research team’s knowledge, this is the first paper in the use of SNFs to report in-depth cost-effectiveness results from multiple perspectives, and one of the few studies for nutrition interventions in low-resource settings that collected primary data to assess uncompensated time.

Including opportunity cost of caregivers’ time was a substantial addition to the total cost in all arms, and especially for the FBFs that required daily preparation before feeding. A 2006 costing report comparing preventative versus recuperative approaches of supplementary feeding found that opportunity cost of beneficiaries’ time (~ 12 h per month per beneficiary) at three critical contact points was not major yet not trivial component (~ 10%) of total cost [34]. This could be an underestimation as women’s relevant time spent at home was not included.

Furthermore, while CSB+ w/oil was the most cost-effective of the four foods from the *program and caregiver perspective* when caregiver time was valued at $0.36/h, RUSF became the most cost-effective when caregiver time was valued at $0.84/h and higher. The valuation of time at $0.36/h was based on the minimum wage of agricultural workers specified under the Burkina Faso labor law, but this value was mandated in 2006 [26] and has not been updated to reflect any changes with national economic growth in more than a decade. When valuing women’s productivity in shea butter production and other female-led activities from a different district of Burkina Faso in 2012, hourly value ranged from $0.57 to $2.27 depending on the segment of the market [35]. A recent literature review described methods used to value time-use in economic evaluations across a broad range of sectors related to health and development in low- and-middle-income contexts. The authors recommended testing an assumption of the value of time between 25 and 75% of the average after-tax wages for activities similar to caregivers’ involvement in this supplementary feeding program [36]. Alternatively, authors recommended conducting a break-even analysis of the opportunity cost of time needed to change the major conclusion [36], which is aligned with the sensitivity analysis in this paper that found $0.84/h to be the break-even point. As different valuation of time changed the conclusion, future research could also consider conducting primary studies to estimate the monetary value for time use, perhaps using the stated preference approach [36]. Results of this paper demonstrate the importance to determine the appropriate hourly value of caregivers’ time in estimating the cost-effectiveness of alternative SNFs in supplementary feeding programs. The findings also highlight the importance of considering program burden on caregivers/recipients when making SNF programming decisions.

According to the research team’s qualitative findings, unpaid distribution committee members asked caregivers to pay a fee of $0.2 (100 CFA) before collecting each monthly ration, even though this practice is prohibited explicitly. The community volunteers seemed to perceive enough burden from the unpaid distribution work and sought partial compensation from caregivers. Because the study’s interview instruments only asked about whether caregivers spent money on transportation, the research team did not collect quantitative data that would allow inclusion of these unexpected monetary costs in the *caregiver* perspective. Instead, the research team accounted for unpaid burden by including the opportunity costs of all program volunteers’ time in the *program* perspective.

For any of the foods tested, paying volunteers the minimum wage of $0.36/h would have added about $19.8/child for distribution committee members and $0.2/child for lead mothers to the total program costs. However, it would reduce burden on the community (directly volunteers and indirectly caregivers by preventing additional charges) and may improve program performance. The $0.2 per monthly ration fee requested by the community volunteers could deter caregivers who were the worst off from coming to collect rations, raising concerns about reaching the most vulnerable recipients. On the other hand, if the program had enforced harsher rules on the distribution committee members to prevent them from charging caregivers, these unpaid community volunteers might be more likely to quit, especially during the busier times of the year, potentially impairing critical last-mile food delivery to recipients. Thus, this paper’s findings raise concerns about the common use of volunteer labor to operate food assistance programs. Future programs should consider providing compensation for all labor.

While CSB+ w/oil, RUSF, and SC+ were similar in effectiveness for primary outcomes of stunting and wasting, CSB+ w/oil was found to be the most cost-effective for achieving these objectives in the blanket supplementary feeding program. The major drivers of cost differences across the three arms were product prices and international freight. The use of more realistic data sources to replace study-incurred costs for product prices and international freight avoided overestimation of cost differences comparing SC+, RUSF, and CSWB w/oil to CSB+ w/oil, with implications for the relative cost-effectiveness rankings between SC+ and RUSF. Doing so would also enhance the generalizability of study results to USAID programmatic settings was also improved. Using realistic procurement price estimates, RUSF had the highest product cost followed by SC+; and SC+ also had the highest supply chain costs. If RUSF or SC+ were preferred for other reasons, such as the reduced preparation burden on caregivers or other programmatic reasons, ways to reduce RUSF and SC+ product costs and SC+ supply chain costs need to be explored to achieve cost-effectiveness comparable to CSB+ w/oil.

The iso-caloric ration size across the four arms is important in interpreting the results. SNF product guidance tables for nutrition programming adopted by Global Nutrition Cluster [37], USAID [38], and World Food Programme (WFP) [39] have specified daily ration sizes to program different types of SNFs for different nutrition purposes. For all FBFs, regardless of formulation, 200 g flour per day is specified for prevention of undernutrition in these tables to account for potential sharing. This is approximately twice as high as the ration provided in each of the three flour arms. In contrast, the recommended daily ration size for ready-to-use food (medium-quantity lipid-based nutrient supplement, LNS-MQ, contains 47–50 g per sachet) is about half of what the research team had used (RUSF contains 100 g per sachet). No specific justification is given in these guidance tables regarding how 200 g/d for the flours was determined appropriate for addressing sharing, nor why there was no apparent need to address sharing in RUSF. According to this paper’s study findings, doubling the recommended ration size for RUSF while halving the recommended ration sizes for SC+ and CSB+ w/oil (that is, providing the same caloric value per ration for all foods) were similarly effective in reducing stunting and wasting. As the research team found product costs and shipping as the largest cost drivers for the arms in this 18-month blanket supplementary feeding program, ration size for each type of SNF would have substantial implications in cost and possibly effectiveness comparisons.

Furthermore, although sharing was more often found among households consuming the flours, a substantial amount of sharing also occurred in the RUSF arm [40]. Thus, this paper’s findings call into question the current suggested ration sizes to program SNFs for the prevention of undernutrition. Future cost-effectiveness research is needed to determine whether sharing would be most cost-effectively addressed through increasing ration size of the specific SNF or by adding general household food assistance (and via which modality: in-kind food commodities, voucher, or cash). If increasing ration size of a specific SNF is indeed more cost-effective to address sharing in a given context, further research is needed to determine the ration size that most cost-effectively incorporates sharing for each type of SNF.

The hypothetical control scenarios provided estimates of cost per case of stunting averted, which allowed the research team to compare the study results with similar estimates from other interventions. Assuming stunting reduction compared to a hypothetical control ranged between 25 and 1 percentage point(s), this paper found that cost per case of stunting averted at 23 months from *program* perspective could range between $506 and $12,659, respectively, in the most cost-effective study arm, CSB+ w/oil.

In comparison, the Rang-Din Nutrition Study reported $1161 USD per case of stunting averted at 18 months for maternal and child supplementation with a LNS-SQ product when compared to a control group with only maternal iron and folic acid supplementation [41]. One should note that the cost per case of stunting averted at 24 month-old for LNS-SQ supplementation became infinitely high in the Rang-Din study as the significant reduction in stunting compared to control diminished as children grew older. Meanwhile, $55 USD per case of stunting prevented at 18 month-old was estimated for a health-facility-based nutrition education program (i.e. no food supplementation) in Peru when compared to control [42]. This would be more cost-effective than if CSB+ w/oil in this study had assumed 25 percentage point stunting reduction compared to control.

Additionally, the hypothetical control scenario results allowed the research team to compare the cost-effectiveness between an arm that was less effective but less expensive (CSWB w/oil), and another arm that was more effective but more expensive (SC+ or RUSF). While CSB+ w/oil remained the most cost-effective option among the four interventions in this analysis, relative cost-effectiveness rankings for the other three choices switched positions depending on the prevalence of stunting in the hypothetical control scenario.

## Conclusions

Evidence generated from the perspectives of multiple stakeholders in this in-depth cost-effectiveness analysis highlighted the importance of caregiver time and unpaid volunteer labor in overall programmatic costs. These different perspectives should be considered when choosing the design of supplementary feeding programs and the products for delivery. Uncompensated time, in particular, could be fundamentally important in determining the sustainability of all feeding programs. In addition, several analytical strategies such as choice of data sources, adjustment in time-use, and hypothetical control scenarios in this multi-perspective cost-effectiveness analysis aimed to improve the validity, comparability, and generalizability of the research findings. Future supplementary feeding program evaluation research should incorporate these considerations in generating cost-effectiveness evidence and refine the field-based techniques required to address them.

## Data Availability

Upon publication of this manuscript, the datasets generated and analyzed during the current study will be made available on the Development Data Library of USAID, found at https://data.usaid.gov. Cost data from accounting and institutional records will not be part of the datasets made available to public.

## Abbreviations

CFA: West African CFA franc, currency of Burkina Faso
CI: Confidence Interval
CSB+ w/oil: Corn Soy Blend Plus with additional fortified vegetable oil
CSWB w/oil: Corn Soy Whey Blend with additional fortified vegetable oil
DHS: Demographic and Health Survey
FAQR: Food Aid Quality Review
FBF: Fortified Blended Flour
ICER: Incremental Cost-Effectiveness Ratio
IRR: Incidence Rate Ratio
LNS: Lipid-based Nutrient Supplement
LTFU: Lost-To-Follow-Up
MT: Metric Ton
OR: Odds Ratio
pp.: Percentage points
RUSF: Ready-to-Use Supplementary Food
SBCC: Social Behavior Change Communication
SC +: Super Cereal Plus
SD: Standard Deviation
SNF: Specialized Nutritious Food
USAID: U.S. Agency for International Development
USD: United States Dollar
WFP: World Food Programme
